# Hysteresis Characteristics and MPI Compensation of Two-Dimensional Piezoelectric Positioning Stage

**DOI:** 10.3390/mi13020321

**Published:** 2022-02-18

**Authors:** Wanqiang Wang, Jiaqi Zhang, Ming Xu, Guojin Chen

**Affiliations:** School of Mechanical Engineering, Hangzhou Dianzi University, Hangzhou 310018, China; wwq@hdu.edu.cn (W.W.); 15988409463@163.com (J.Z.); chenguojin@163.com (G.C.)

**Keywords:** piezoelectric positioning stage, hysteresis, MPI, compensation, fitting coefficient

## Abstract

Piezoelectric positioning stage is the core component of precision positioning system and advanced instrument. Its hysteresis characteristics, especially rate-dependent characteristics, are the main factors affecting the positioning or control accuracy. The multi-slope Prandtl–Ishlinskii (MPI) based hysteresis modeling and compensation experiments of two-dimensional piezoelectric positioning stage are discussed. The impact of the driving voltage amplitude and frequency on the hysteresis characteristics of uniaxial piezoelectric actuator in the piezoelectric positioning stage are studied, especially the influence of variable-frequency voltages on the output displacement of a piezoelectric actuator. The MPI compensation control of two-dimensional piezoelectric positioning stage is carried out, and the fitting coefficient *R*^2^ is proposed to evaluate the hysteresis compensation accuracy of MPI model. Under the full range driving voltage of 20~120 V, the fitting coefficient reaches more than 99.6%. The experiments of feedforward compensation and feedback compensation are implemented. Having applied the composite triangular-wave signal, the average absolute displacement error of the piezoelectric actuator is 0.1192 μm, as well as the mean square error 0.2949 μm. It demonstrates that the MPI model is effective against hysteresis for two-dimensional piezoelectric positioning stage.

## 1. Introduction

The piezoelectric positioning stage is widely used in the field of precision instruments because of its advantages of precise positioning and rapid response [1,2,3], such as the precision positioning system [3], atomic force microscope [4,5], piezoelectric tilt mirror [6], piezoelectric MR image guided robotic system [7], microscopy stage [8], space applications [9], vibration-assisted micro-milling [10], etc. The piezoelectric ceramic actuator is the core component of the piezoelectric positioning platform, but its strong nonlinear characteristics, especially hysteresis nonlinearity, are the major hindrance affecting the positioning or control accuracy of piezoelectric positioning stage [1,2,3].

To reduce the influence of hysteresis nonlinearity of piezoelectric ceramics and improve the control accuracy, it is usually necessary to establish a model to describe the hysteresis nonlinearity and adopt strategies for compensation control [11]. In a large number of studies, feedforward compensation is a promising scheme [1,12,13,14]. The piezoelectric hysteresis model is established to track the actual hysteresis curve, and then the feedforward method is chosen to compensate for the hysteresis error. In the hysteresis feedforward compensation for piezoelectric ceramics, the hysteresis model generally requires reversibility [2], which can theoretically eliminate the influence of hysteresis nonlinearity used in the feedforward control, so as to improve the control accuracy of micro displacement positioning or driving system.

There are two kinds of nonlinear hysteresis modeling methods, the constitutive method [15] and phenomenological method [16,17,18,19,20]. The constitutive model is deduced based on the inherent constitutive relationship of piezoelectric ceramics, but the establishment process is difficult due to the complex internal characteristics of the hysteresis phenomenon [1,15]. The phenomenological method approximates the measured hysteresis curve by directly establishing mathematical models [16,17,18,19,20], such as Bouc–Wen (B–W) model [21], KP model [22], Preisach model [23], Prandtl–Ishlinskii (PI) model [24,25,26,27,28,29,30,31], etc. Among them, the PI model has attracted extensive attention because it is easy to implement the inverse model [24]. The PI model contains two basic operators, play operator and stop operator, which are obtained by scaling and superimposing the operators through appropriate weight functions. Since each play (or stop) operator is geometrically represented as a parallelogram with central symmetry, the PI model after operator superposition also has symmetry [30,31]. In fact, a variety of hysteresis curves, including piezoelectric actuators, show asymmetry, resulting in a large error of the PI model in describing such hysteresis curves [1,31].

To make the PI model have the ability to characterize the asymmetric hysteresis curve, scholars have attempted to modify it as follows: (1) the memoryless function of the PI model is modified into the form of a hysteresis curve, which is superimposed onto the memory function to change the symmetry of the model. For example, Gu et al. [28] proposed to change the memoryless function of the third-order polynomial. Although this method changes the overall model symmetry and improves the modeling accuracy to a certain extent, it lacks a detailed description of the measured hysteresis curve. (2) Change the symmetry of memory function, more specifically, change the symmetry of a single operator. Al janaideh et al. [29] proposed a GPI model based on a generalized play operator, which is composed of two user-defined envelopes. Although this method changes the symmetry of the operator, the envelope of the operator needs to be selected according to experience. Li et al. [30] divided the operator into upper and lower parts to change the symmetry of the operator and establish the SPI model. However, this method, by changing the symmetry of a single operator, may cause discontinuous output at the turning point of the hysteresis curve. Taking the piezoelectric ceramic PZT-5H as an example, the authors proposed an improved multi-slope PI model, namely the MPI model, which uses polynomials to correct the symmetry of PI operators. The simulation and experiments show that the error of MPI model is significantly lower than that of the classical PI model [31].

According to the difference between the hysteresis characteristic curves of the two-dimensional piezoelectric positioning stage and PZT-5H, the MPI model is further modified and simplified. The Section 2 introduces the experimental system and discusses the hysteresis characteristics of the two-dimensional piezoelectric positioning platform. In the Section 3, the amendments of the MPI model have been made towards the purpose of simplification. In the Section 4, the hybrid controller composed of feedforward and feedback compensation is designed, and the MPI hysteresis compensation performance is demonstrated by experiments.

## 2. Hysteresis of Piezoelectrics

### 2.1. Experimental System

**Figure 1:**
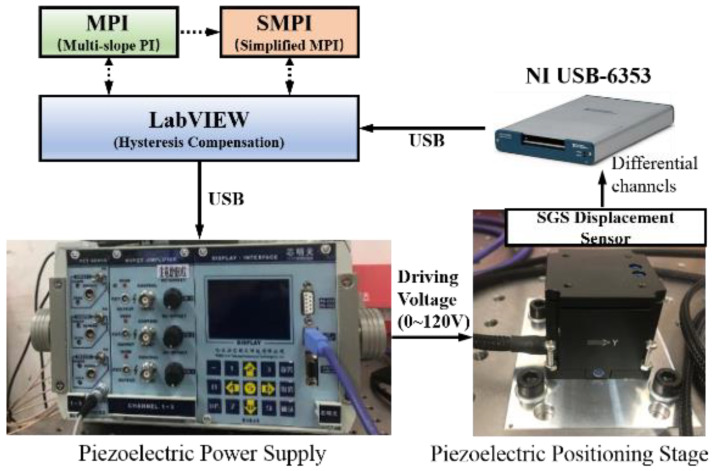
Experimental system.

As shown in Figure 1, the experimental system mainly includes a piezoelectric positioning stage, a piezoelectric power supply, and a data acquisition device. The piezoelectric positioning platform P11.XY100 (referred to as P11) with built-in SGS (resistance strain gauge sensor) displacement sensors is produced by Harbin Core Tomorrow Science & Technology Co., Ltd., Harbin, China, which is a two-dimensional piezoelectric positioning platform composed of two one-dimensional piezoelectric ceramic actuators. It adopts a frictionless flexible hinge as the chain guide mechanism, which can realize the displacement output in X and Y directions. The driving voltage ranges from 0 V to 120 V, as well as the nominal stroke 0 μm~80 μm. The maximum stroke is 110 μm, and the corresponding driving voltage range is −20 V to 150 V. The piezoelectric power supply is E18.i3 produced by Core Tomorrow Co., Ltd., which can realize 3-channel control, 24 bit ± 10 V D/A and 16 bit ±10 V A/D conversion accuracy. The communication interface includes RS-232/422 and USB, and the voltage control range is 0 V–150 V. The SGS displacement sensing signal built in the piezoelectric actuator is collected through the differential channel of NI USB-6353, and can then be employed by MPI (Multi-slope PI) and SMPI (Simplified MPI) hysteresis compensation methods in LabVIEW.

### 2.2. Voltage Amplitude on Piezoelectric Hysteresis

The X- and Y-axis displacements of the two-dimensional piezoelectric positioning platform P11 are independent of each other. Taking the single-axis displacement as an example, the influence of the amplitude and frequency of the driving voltage on the hysteresis curve is analyzed, which is the basis of the design for the compensation algorithm.

**Figure 2:**
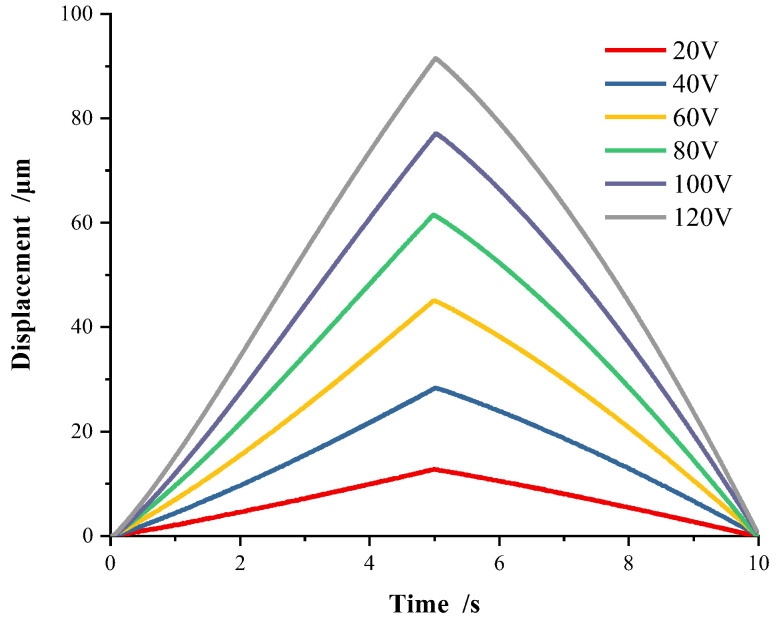
Hysteresis on variable voltage amplitudes (0.1 Hz).

The hysteresis characteristics of the two-dimensional piezoelectric positioning platform under the amplitude variation on driving voltage are firstly considered. Since the driving voltage range is 0–120 V, the triangular-wave voltages with amplitude of 20 V, 40 V, 60 V, 80 V, 100 V, and 120 V at frequency 0.1 Hz are applied to the piezoelectric stage. The piezoelectric hysteresis curves under variable voltage amplitudes are obtained, as shown in Figure 2.

As shown in Figure 2, the obvious hysteresis nonlinear occurs, in where each hysteresis curve in the voltage rise stage and fall stage is asymmetric on variable voltage amplitudes.

### 2.3. Voltage Frequency on Piezoelectric Hysteresis

**Figure 3:**
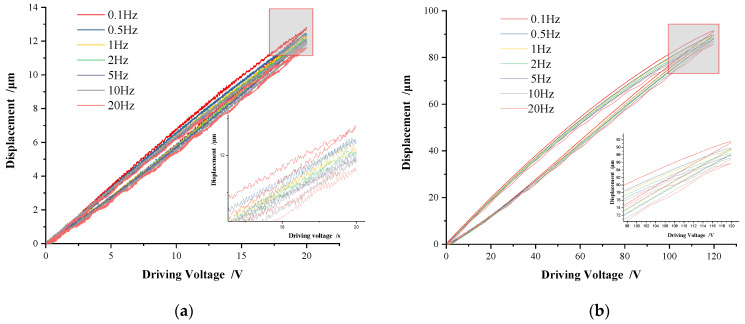
Hysteresis curves on variable voltage frequencies. (**a**) 20 V, (**b**) 120 V.

To study the hysteresis characteristic of the driving voltage frequency of the piezoelectric positioning platform, triangular-wave signals were set to amplitude 20 V and frequency at 0.1 Hz, 0.5 Hz, 1 Hz, 2 Hz, 5 Hz, 10 Hz, and 20 Hz, respectively; the piezoelectric hysteresis curves were obtained as shown in Figure 3a. Similarly, when the voltage amplitude was 120 V, the hysteresis curves were obtained as shown in Figure 3b.

As can be seen from Figure 3, no matter what the frequency of the driving voltage is, the displacement and the driving voltage show a highly linear relationship in the voltage step-up stage, which is closely related to the structural principle of the piezoelectric positioning platform, that is, the preloading measure is taken to weaken its hysteresis characteristics. In the voltage step-down stage, the piezoelectric actuator is generally in a non-working state with visible hysteresis, as shown in Figure 3. In addition, it can also be seen that the greater the frequency of the driving voltage, the more obvious the hysteresis, indicating the rate-dependent characteristics of the piezoelectric positioning platform [17,18,19].

### 2.4. Voltage on Piezoelectric Hysteresis

**Figure 4:**
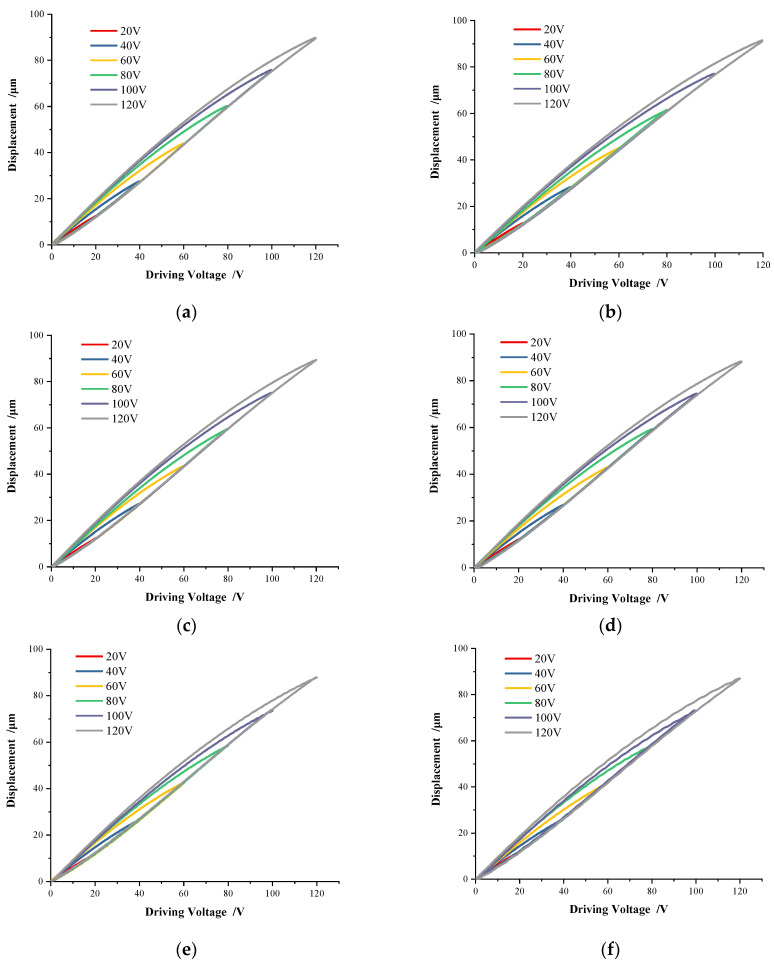

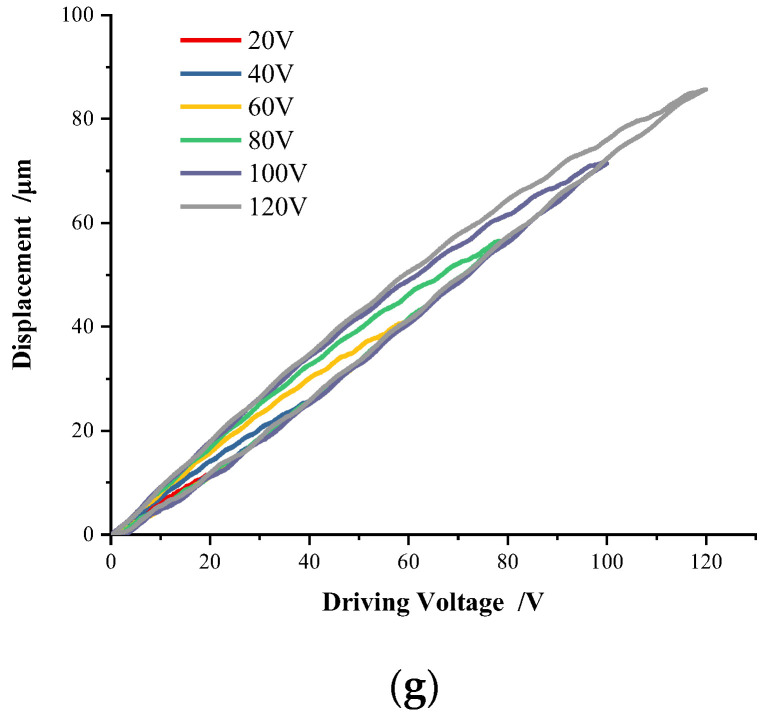
Hysteresis on variable voltages. (**a**) 0.1 Hz; (**b**) 0.5 Hz; (**c**) 1 Hz; (**d**) 2 Hz; (**e**) 5 Hz; (**f**) 10 Hz; (**g**) 20 Hz.

Furthermore, the piezoelectric hysteresis curves under variable frequency and amplitude voltage are shown in Figure 4.

It can be seen that the hysteresis curves of voltages with different amplitudes and frequencies show high linearity when in the voltage step-up stage, which is consistent with the conclusion drawn in Figure 3. At the same frequency, the influence of voltage amplitude on the hysteresis curve is straightforward. The larger the voltage amplitude is, the larger the hysteresis loop is. Meanwhile, the influence of variable voltage frequency on the hysteresis loop is not visible. Only when the frequency is high (as shown in Figure 4f,g), the hysteresis curve will oscillate obviously.

**Figure 5:**
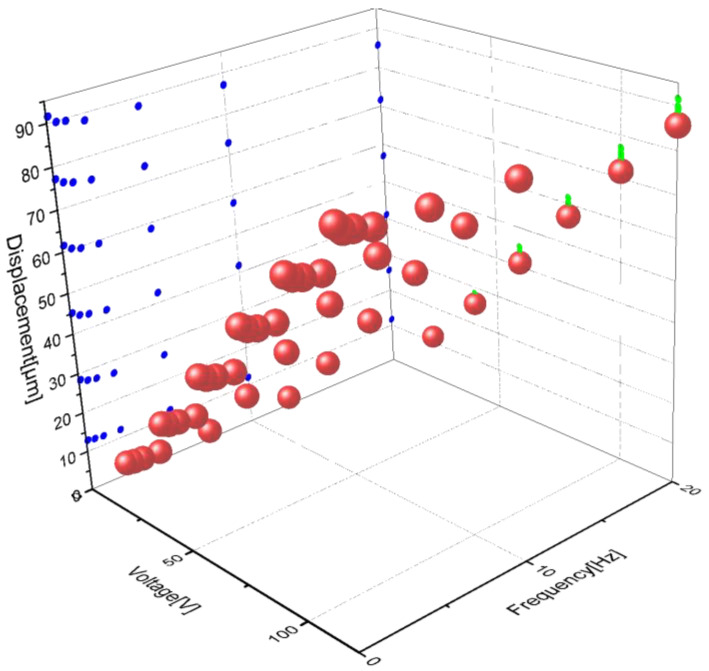
Peak Displacement on variable voltages.

The peak displacement values of each group of hysteresis curves in Figure 4 can be drawn in Figure 5, where the X-axis represents the voltage amplitude, the Y-axis represents the voltage frequency, and the Z-axis represents the peak displacement value under the corresponding voltage and frequency.

**Figure 6:**
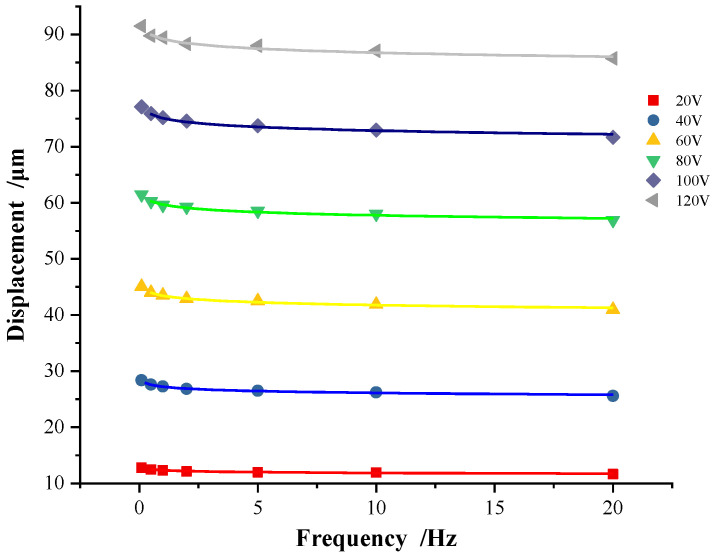
Relationship between peak displacement and driving voltage frequency.

The peak displacement values in Figure 5 are transformed into another form, as shown in Figure 6, where each curve represents the fitting result of all discrete peak displacement values at variable frequencies. As shown in Figure 6, the peak displacement of the piezoelectric actuator is independent of the frequency in the most frequency ranges. Only in the very low-frequency range, the peak displacement decreases with the increase in frequency.

To further explore the relationship between peak displacement and driving voltage frequency, the mathematical model shown in Equation (1) is proposed.

(1) $$X_{p}=af^{b}$$

where *Xp* is the peak displacement value, *f* is the voltage frequency, *a* and *b* are the undetermined parameters.

For the discrete points of the peak displacement value under six groups of voltage amplitude, the parameters are determined by Equation (1) as shown in Table 1.

It can be seen from Table 1 that the value of parameter ‘*a*’ changes uniformly with the voltage amplitude, while the value of parameter ‘*b*’ is basically unchanged. The values of parameter ‘*a*’ are arranged in equal difference, which shows the linear relationship between the peak displacement and the voltage amplitude. For each variable voltage frequency, the value of *f^b^* at 40 V (*b* = −0.018) is almost equivalent to that at 120 V (*b* = −0.012). The relative error ratio, defined as (|*f^b^* (40 V) − *f^b^* (120 V)|)/*f^b^* (40 V), at each frequency is within 1.8%. Compared to the parameter ‘*a*’, this change of parameter ‘*b*’ is not remarkable. Since the value of parameter ‘*b*’ is basically unchanged, the change of voltage amplitude basically does not affect the shape of the hysteresis curve.

## 3. MPI Hysteresis Compensation

For the piezoelectric positioning stage P11 and the piezoelectric ceramics PZT-5H in [31], the hysteresis curve is significantly different, which is mainly reflected in that P11 has higher linear characteristics in the process of voltage increase, that is, because the P11 is composed of preloaded high-performance piezoelectric ceramics. However, the PZT-5H is an ordinary piezoelectric ceramic without preload, which presents a nonlinear hysteresis curve in the process of voltage increases [31]. Although the voltage increase hysteresis curves are different, both show obvious hysteresis characteristics in the process of voltage reduction. In addition, the driving voltage amplitude range of P11 is 0 V–120 V, while the driving voltage amplitude range of PZT-5H is 0 V–150 V. When applying the MPI model to the hysteresis modeling on P11, the model parameters need to be modified. The modified MPI model expression is as follows:

(2) $$\left\{ \begin{array}{l} f_{k}\left(0\right)=\max\{u\left(0\right)-r_{k},\min\left\{\min\left[1.2u\left(0\right),0.8u\left(0\right)+0.2V_{p}-0.6r_{k}\right],\;y\;(0)\right\}\},\\ f_{k}\left(t\right)=\max\{u(t)-r,\min\left\{\min\left[1.2u\left(t\right),0.8u\left(t\right)+0.2V_{p}-0.6r_{k}\right],\;y\;(t\text{-}T)\right\}\},t>0. \end{array}\right.$$

where *V_p_* is the driving voltage amplitude, *f_k_* (*t*) is the play operator with threshold *r_k_*, *u*(*t*) is the driving voltage applied to the piezoelectric ceramics, and *T* is the sampling period.

The model described in Equation (2) was used to fit multiple groups of piezoelectric hysteresis curves with different amplitudes and frequencies. The preliminarily selected thresholds *r_k_* are arranged at equal intervals. The thresholds of different voltage amplitudes are different, and they are selected according to the following rules: 13 groups of thresholds (0, 10, …, 120) with voltage amplitude of 120 V are selected; for the threshold with a voltage amplitude of 20 V–120 V, select (0, 10, …, 100) × *V_p_*/100, 11 groups in total. The model parameter identification adopts the quadratic programming algorithm.

**Figure 7:**
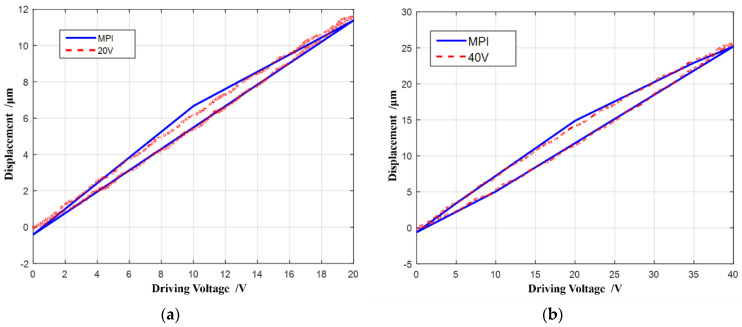

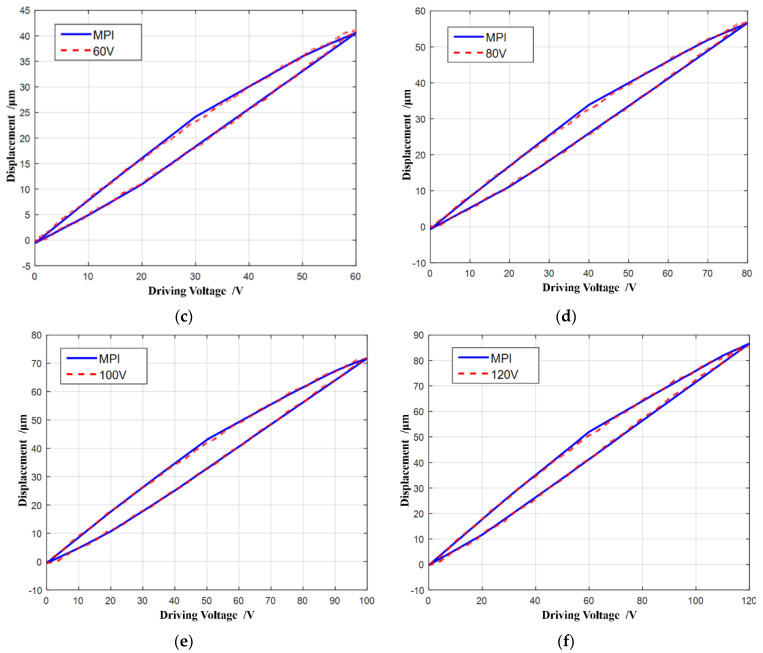
MPI model accuracies on variable voltage amplitudes. (**a**) 20 V; (**b**) 40 V; (**c**) 60 V; (**d**) 80 V; (**e**) 100 V; (**f**) 120 V.

When the driving voltage frequency is 20 Hz and the amplitude is 20 V, 40 V, 60 V, 80 V, 100 V, or 120 V, the fitting degree of the MPI hysteresis model to each driving voltage hysteresis curve is obtained, as shown in Figure 7.

As shown in Figure 7, the MPI model curve can obtain very high fitting accuracy under all driving voltages. During the voltage rise phase, the MPI curve almost coincides with the hysteresis curve under all driving voltages. There is only a small error in the middle of the driving voltage decrease, that is, the place with the largest hysteresis.

The maximum absolute error, average absolute error, and mean square error are used to evaluate the accuracy of MPI model, as shown in Table 2. It can be seen that the MPI model can characterize the hysteresis curve of the piezoelectric positioning platform with high precision under different driving voltages. The mean absolute error and mean square error enlarge with the increase of driving voltage. Except for voltage 120 V, the mean absolute error and mean square error are less than 0.3 μm and 0.4 μm, showing very high model accuracy.

Furthermore, a coefficient *R*^2^ representing the fitting degree of excellence is proposed to describe the compensation accuracy of the MPI model, and its expression is as follows:

(3) $$R^{2}=1-\frac{SSE}{SST}=1-\frac{\sum_{i=1}^{n}{\left(y_{i}-{\hat{y}}_{i}\right)}^{2}}{\sum_{i=1}^{n}{\left(y_{i}-{\bar{y}}_{i}\right)}^{2}}$$

where *SSE* is the sum of squares of residuals, *SST* is the sum of total squares. *n* is the number of data points, and set *n* = 2000 according to the data acquisition cycle. *i* is an integer in (1, *n*). *y_i_* is the *i*th data point of hysteresis curve. ${\hat{y}}_{i}$ is the *i*th data point obtained according to MPI model. $\bar{y}$ is the mean value of all data points of the actual hysteresis curve.

According to Figure 7 and Table 2 and Table 3, it can be seen that the MPI model has an excellent fitting performance on the hysteresis curve with voltage frequency of 20 Hz and amplitude of 20 V, 40 V, 60 V, 80 V, 100 V, and 120 V, respectively. The fitting degree of excellence is more than 0.996. The error of the MPI model is mainly concentrated on the middle region of the driving voltage decrease, that is, the region with the most obvious hysteresis phenomenon.

**Figure 8:**
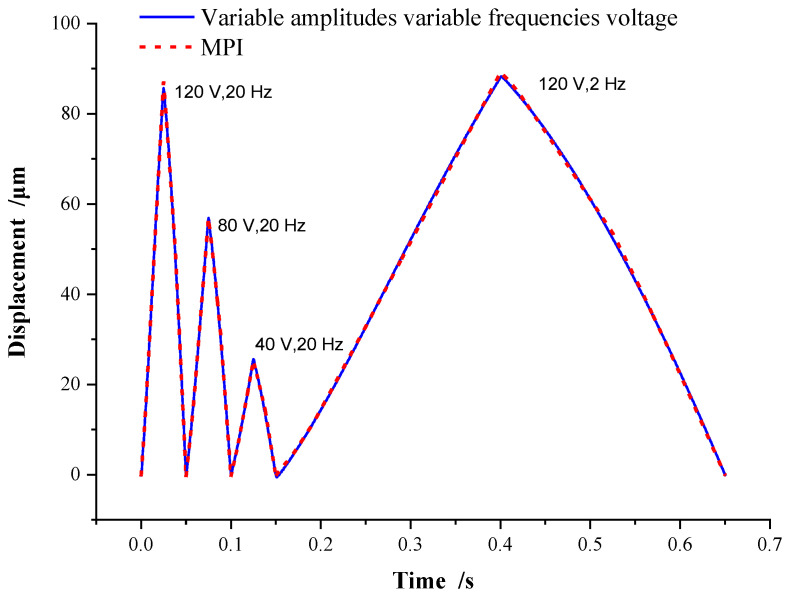
MPI accuracy on variable amplitudes variable frequencies voltage.

**Figure 9:**
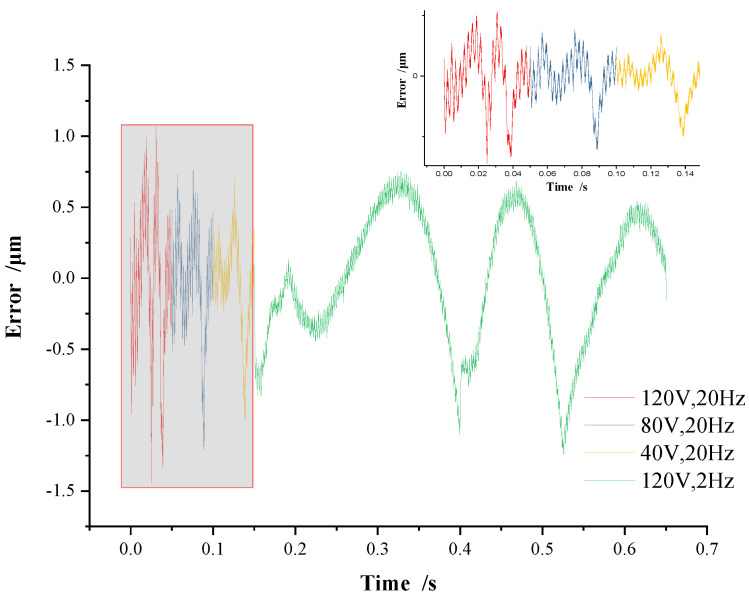
MPI error on variable amplitudes variable frequencies voltage.

To better prove the accuracy and adaptability of the MPI model, a group of voltage inputs with variable amplitude and variable frequency is designed, which is composed of continuous triangular-wave voltage signals with an amplitude of 120 V, 80 V, and 40 V at a frequency of 20 Hz, and an amplitude of 120 V at a frequency 2 Hz, respectively. The displacement and fitting errors of the MPI model are shown in Figure 8 and Figure 9.

As shown in Figure 8, under the variable amplitudes variable frequencies voltage, the curve of the MPI model almost coincides with the piezoelectric hysteresis curve, and the peak displacement under each voltage is consistent with Figure 7. The error of the MPI model is shown in Figure 9, where the error is basically within ±1 μm. It demonstrates that the MPI model can achieve high accuracy and adaptability, and can be adopted to the hysteresis curve of the piezoelectric actuator with different voltage amplitude and frequency combinations. In Figure 9, the hysteresis error curves of the MPI model under the conditions of 120 V at a frequency of 20 Hz and 120 V at a frequency of 2 Hz are similar, indicating that the voltage frequency has little effect on the accuracy of the MPI model under the same voltage amplitude. This is also consistent with the conclusion obtained in Figure 6.

Since the influence of voltage frequency on hysteresis is much less than that caused by the voltage amplitude, the displacement peak parameter is introduced into the MPI model. According to Equation (2), a SMPI (Simplified MPI) model suitable for different voltage amplitude conditions is proposed based on the MPI model with a voltage amplitude of 120 V at frequency 20 Hz. The SMPI expression is described as follows:

(4) $$y(t)=\frac{X_{pi}}{X_{p120}}\sum_{k=0}^{n}\omega_{k}\max\{u\left(t\right)-r_{k},\min\left\{\min\left[1.2u\left(t\right),0.8u\left(t\right)+0.2V_{p}-0.6r_{k}\right],\;y\;(t\text{-}T)\right\}\}$$

where *X*_*p*120_ is the peak displacement value at voltage amplitude 120 V, and *X_pi_* represents the peak displacement value at any voltage amplitude in the range of 0 V to 120 V.

**Figure 10:**
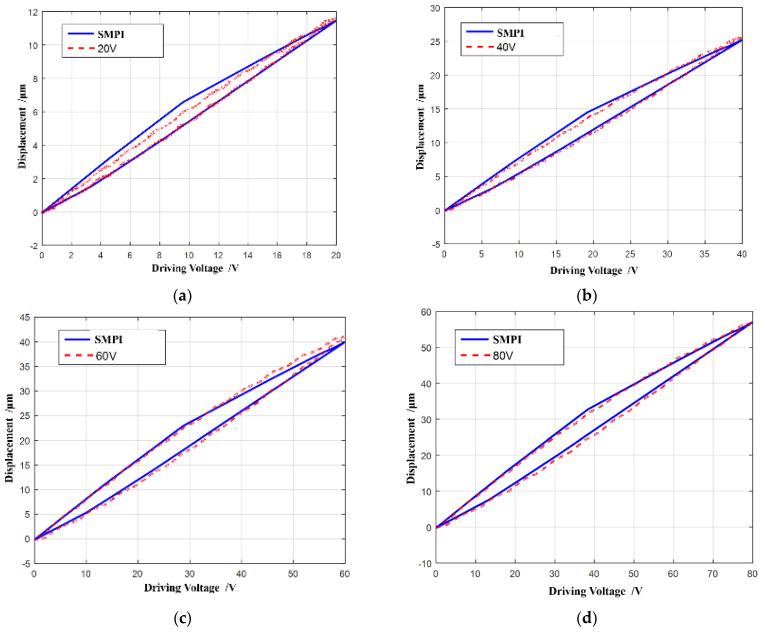

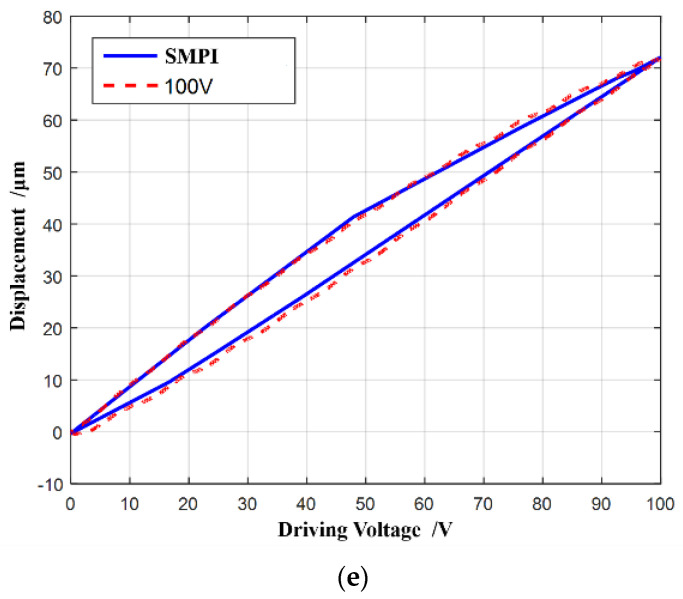
SMPI accuracies on variable voltage amplitudes (20 Hz). (**a**) 20 V; (**b**) 40 V; (**c**) 60 V; (**d**) 80 V; (**e**) 100 V.

The SMPI model is used to fit the piezoelectric hysteresis of variable voltage amplitudes at frequency 20 Hz, and the results are shown in Figure 10.

As shown in Figure 10, the SMPI model curve can also almost coincide with the hysteresis curve under each voltage. Similar to the MPI model, the accuracy and adaptability of the SMPI model are also evaluated by the error and fitting coefficient, as shown in Table 4 and Table 5.

Comparing Table 2 and Table 4, it can be seen that the model error of SMPI increases a little compared with MPI, but the mean absolute error is within 0.75 μm and the mean square error is within 0.85 μm, which still shows a very high hysteresis fitting accuracy. In other words, the SMPI model can greatly simplify the complexity of the hysteresis model and still ensure the fitting accuracy, showing good adaptability. It can also be seen from Table 5 that the fitting coefficients of the SMPI model are more than 0.99, which is equivalent to that of the MPI model.

## 4. Experiments and Discussions

**Figure 11:**
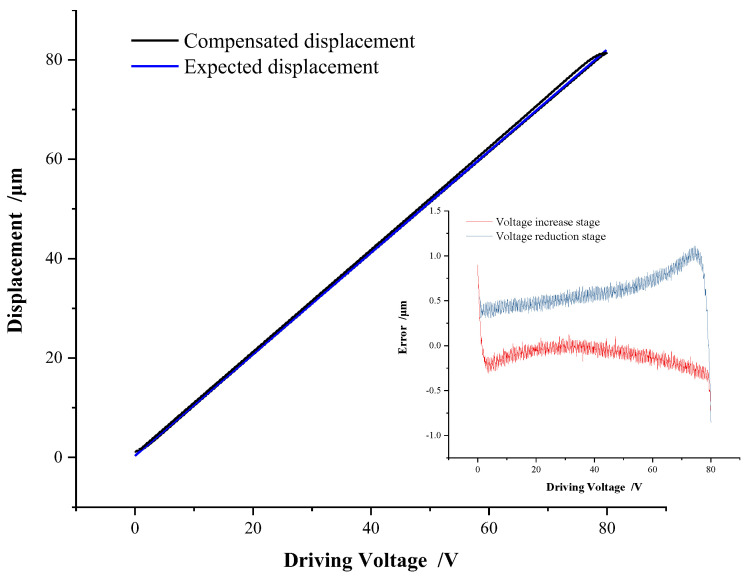
SMPI feed-forward compensation.

The SMPI model can ensure high accuracy for piezoelectric hysteresis with a simple model structure and inverse model accessibility, which is very suitable for hysteresis compensation control. Taking the triangular-wave voltage signal with an amplitude of 80 V at a frequency of 1 Hz as an example, the feedforward hysteresis compensation performance of the SMPI model is demonstrated by experiment, as shown in Figure 11.

It can be seen from Figure 11 that the displacement of the compensated piezoelectric stage almost coincides with the expected displacement curve, and the compensated displacement shows a highly linear relationship with the driving voltage. The error in the voltage increase phase is basically maintained at about 0.3 μm, while the error in the voltage decrease phase is slightly larger than that in the voltage rise phase because the piezoelectric actuator is in the non-preload condition. In the voltage decrease phase, the error is about 0.5 μm most of the time, and the maximum error is maintained within 1 μm. Considering the whole stage of triangular-wave voltage, the average absolute error is 0.0485 μm, and the mean square error is 0.1201 μm. The SMPI model and its feedforward control demonstrate a fairly high compensation accuracy.

**Figure 12:**
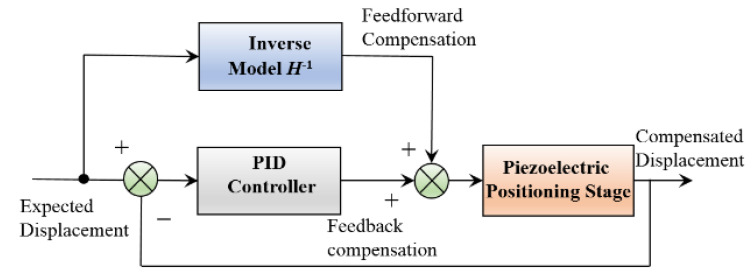
Scheme of hysteresis compound compensation.

The feedforward controller is mainly used to compensate for hysteresis nonlinearity. When the system encounters uncertain disturbances, the feedforward controller cannot adapt in time. Therefore, the feedforward and feedback algorithm are generally combined to form a compound controller [13,14], which can compensate for the hysteresis and eliminate the system disturbance at the same time, as shown in Figure 12.

The PID method is simple and practical and is often chosen to be used in the compensation control of the piezoelectric stage [1,2,14]. The tuning of PID parameters follows the Ziegler–Nichol method and the comprehensive setting method based on the error performance index, especially for the middle part of the hysteresis curve, because the error of this part is relatively large in the whole hysteresis stage.

The hybrid driving voltage is designed, including five groups of variable amplitude variable frequency driving voltage, as shown in Table 6, to verify the compound compensation strategy.

**Figure 13:**
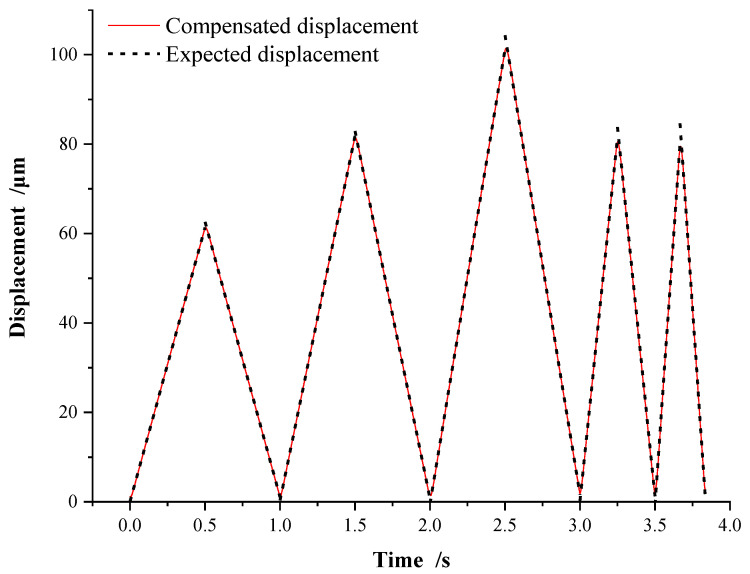
Hysteresis compound compensation.

Figure 13 shows the hysteresis compensation performance of five groups of different expected peak displacements and frequencies after adopting the compound compensation scheme shown in Figure 12. The compensated displacement curve coincides with the expected displacement curve at each peak displacement and frequency. The hysteresis compensation method using the SMPI model can track the desired displacement with high precision.

**Figure 14:**
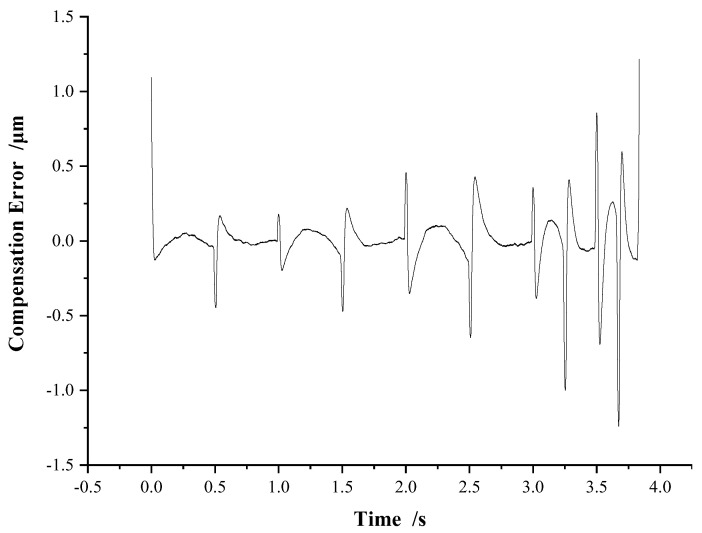
Error of hysteresis compound compensation.

As shown in Figure 14, the compensation error is basically within ±0.5 μm. The error spike appears only at the peak and valley points of displacement, which is caused by the sudden change in motion direction of the piezoelectric actuator. The average absolute error between the actual displacement and the expected displacement of P11 after hysteresis compound compensation is 0.1192 μm, and the mean square error is 0.2949 μm. The results show that the compound control method has high hysteresis compensation performance and excellent stability.

## 5. Conclusions

The relationship between the peak displacement of the piezoelectric actuator and the driving voltage frequency is established. The experiment shows that the peak displacement is linear with the voltage amplitude. The change in voltage amplitude basically does not affect the shape of the hysteresis curve, and the peak displacement is basically independent of frequency. The fitting coefficient *R*^2^ is proposed to characterize the hysteresis compensation accuracy of the MPI model, which reaches more than 99.6% under the driving voltage of 20 V~120 V. Under the action of composite triangular-wave signal, the average absolute error is 0.1192 μm, and the mean square error is 0.2949 μm, which demonstrates that the SMPI model has high accuracy for hysteresis compensation in the piezoelectric ceramic actuator.

## Figures and Tables

**Table 1:** Fitting parameters.

Voltage Amplitude (V)	*a*	*b*
20	12.306	−0.017
40	27.235	−0.018
60	43.448	−0.017
80	59.669	−0.014
100	75.08	−0.013
120	89.189	−0.012

**Table 2:** MPI model accuracies on variable voltage amplitudes.

Driving Voltage (20 Hz Triangle-Wave)	Maximum Absolute Error (μm)	Mean Absolute Error (μm)	Mean Square Error (μm)
20 V	0.4215	0.1184	0.1451
40 V	1.0007	0.2306	0.3164
60 V	0.9785	0.2554	0.3217
80 V	1.2104	0.2753	0.3611
100 V	1.0687	0.2941	0.3724
120 V	1.4441	0.4002	0.5053

**Table 3:** Fitting degree of excellence in MPI.

Driving Voltage	20 V	40 V	60 V	80 V	100 V	120 V
*R* ^2^	0.9960	0.9983	0.9993	0.9996	0.9997	0.9996

**Table 4:** SMPI model accuracies on variable voltage amplitudes.

Driving Voltage (20 Hz Triangle-Wave)	Maximum Absolute Error (μm)	Mean Absolute Error (μm)	Mean Square Error (μm)
20 V	0.7348	0.1936	0.2692
40 V	1.0278	0.3300	0.3986
60 V	1.7215	0.5507	0.6678
80 V	1.5595	0.6115	0.7322
100 V	1.7403	0.7126	0.8491

**Table 5:** Fitting degree of excellence in SMPI.

Driving Voltage	20 V	40 V	60 V	80 V	100 V
*R* ^2^	0.9939	0.9973	0.9970	0.9982	0.9985

**Table 6:** Parameters of hysteresis compound compensation.

Group	1	2	3	4	5
Expected Displacement (μm)	60	80	100	80	80
Frequency (Hz)	1	1	1	2	3

## Data Availability

The data presented in this study are available on request from the corresponding author.
